# Developing a strategy for identifying recommendations prioritized for implementation in the Colombian health system

**DOI:** 10.1186/s12961-026-01465-6

**Published:** 2026-09-10

**Authors:** Marcela Vélez, Pamela Velásquez-Salazar, Claudia Yaneth Vera-Giraldo, Juan Carlos Velásquez-Correa, Julián Santiago Franco, Luz Helena Lugo-Agudelo, Viviana Vélez-Marín, Christine Fahim, Robert Marten, Sonam Yangchen, Sharon Straus

**Affiliations:** 1 Faculty of Medicine, University of Antioquia, Cra 51d #62-29, Medellín, Antioquia Colombia; 2 Knowledge Translation Program, Li Ka Shing Knowledge Institute, St. Michael’s Hospital, 30 Bond Street, Toronto, ON M5B 1W8 Canada; 3 Alliance for Health Policy and Systems Research, 20 Avenue Appia, 1211 Geneva, Switzerland

**Keywords:** Implementation, Clinical practice guidelines, Health systems guidelines, Priorities

## Abstract

**Background:**

Health systems guidelines (HSGs) developed by the WHO and clinical practice guidelines (CPGs) developed by the Colombian Ministry of Health can potentially support national and subnational policy-development processes. Barriers to implementation encountered when making necessary changes to the Colombian health system led to our research question: What prioritized recommendations are pending implementation in Colombia, and which require changes in health system arrangements?

**Methods:**

A document analysis of all 58 Colombian CPGs and all 14 WHO HSG documents was performed in April 2020. We extracted information about recommendations prioritized on the CPGs/HSGs, updated status (documents were outdated if 5 years had elapsed since publication, and close to outdated if they were in the fifth year since publication), and the strength and direction of the recommendation as graded by the guideline development group. Recommendations were evaluated by two researchers to determine whether their implementation required making changes to the health system. Those changes were classified into three dimensions: governance, financial and delivery arrangements.

**Results:**

In the 71 guidelines, 958 recommendations were identified. A total of 393 (41.0%) were updated, 425 (44.4%) were outdated and 140 (14.6%) were close to outdated. Regarding the strength and direction of the recommendations, 540 were strong (56.4%), 189 were weak (19.7%) and 173 were suggested or recommended in a specific context (18.1%). Most of the recommendations were in favour of the intervention (*n* = 861, 89.9%), and only 97 were against the intervention (10.1%). Among the 363 recommendations from the WHO HSGs, 244 (67.2%) had not been implemented in Colombia. Of 958 recommendations, 518 (54.1%) needed a health system arrangement for their implementation: 259 in governance, 102 in financial and 503 in delivery.

**Conclusions:**

We identified 518 recommendations that require change in Colombian health system arrangements. The most common change was related to availability of care, given that many of the recommendations refer to technologies/services approved and publicly funded but not widely available or accessible to different population groups. Further research may evaluate whether this strategy helps to define priorities for implementation.

## Background

Implementation of clinical practice and health systems guidelines is a common challenge for health systems. A systematic review of studies in which guideline use was evaluated revealed that adoption and adherence were low even when awareness of and agreement with guidelines among target users were high [1]. Evidence indicates that it takes up to 17 years to translate 14% of published research into practice [2]. In response to the challenge of knowledge translation, guideline development has increasingly focused on planning how to effectively implement recommendations in the health system [3, 4], and developing mechanisms and strategies that help to identify which ones are a priority for implementation from a large set of evidence-based recommendations [5, 6].

Colombia has a social security health system of managed competition, where private institutions intermediate the provision of healthcare services (included in a benefits plan) between a unique public fund and private and public healthcare providers. Between 2008 and 2016, the Colombian Ministry of Health (CMoH) in an effort to reduce unexplained variations in medical care and efficient management of resources [7], developed 58 national clinical practice guidelines (CPGs) for prioritized health conditions (Table 1). Some of the criteria used to prioritize CPG development were whether they addressed diseases of interest for public health, high-frequency conditions, diseases that represent a significant social and economic burden, and diseases or conditions for which interventions might have an immediate positive impact [7]. In addition to economic resources, the CPGs have mobilized the research capacity of many different institutions (e.g. universities, hospitals, professionals and patients’ organizations), who used the highest methodological standards to develop these national guidelines. The CMoH guidelines are considered part of the health system’s regulations and should be used to inform policy, planning, evaluation and quality improvement [8–10]. The implementation of CPGs has enhanced the Colombian health system in different ways, for instance, it has incentivized updates to the health benefits plan [11], and led to improvements in health, for instance, it has helped to decrease maternal mortality rates [12]. However, implementing approximately 3500 recommendations is an arduous process for the CMoH and health authorities.

**Table 1:** Guidelines reviewed (WHO and CMoH)

No.	CPG name in Spanish	CPG name in English	Field	Year of publication or update
1	Guía de la OMS sobre políticas de salud y apoyo al sistema para optimizar los programas comunitarios de trabajadores de salud	WHO guidelines on health policy and system support to optimize community health worker programmes	Health systems	Published 2018
2	Recomendaciones de la OMS sobre registros domiciliarios para la salud materna, neonatal e infantil	WHO recommendations on home-based records for maternal, newborn and child health	Health systems	Published 2018
3	Guía para la prevención y el control de Enterobacteriaceae resistente a carbapenem, *Acinetobacter baumannii* y *Pseudomonas aeruginosa* en instalaciones de salud	Guidelines for the prevention and control of carbapenem-resistant Enterobacteriaceae, *Acinetobacter baumannii* and *Pseudomonas aeruginosa* in health care facilities	Health systems	Published 2017
4	Rehabilitación en sistemas de salud	Rehabilitation in health systems	Health systems	Published 2017
5	Guía de la OMS sobre atención integrada para personas mayores (ICOPE)	WHO guidelines on integrated care for older people (ICOPE)	Health systems	Published 2017
6	Guía global sobre la prevención de la infección del sitio quirúrgico	Global guidelines on the prevention of surgical site infection	Health systems	Published 2016
7	Guía sobre los componentes básicos de los programas de prevención y control de infecciones a nivel nacional e internacional de atención médica aguda	Guidelines on core components of infection prevention and control programmes at the national and acute healthcare facility level	Health systems	Published 2016
8	Guía de la OMS sobre el uso de jeringas de seguridad para inyecciones intramusculares, intradérmicas y subcutáneas en entornos de atención médica	WHO guideline on the use of safety-engineered syringes for intramuscular, intradermal and subcutaneous injections in healthcare settings	Health systems	Published 2016
9	Las funciones de los trabajadores de salud en la prestación de servicios de aborto seguro y anticoncepción postaborto	Health worker roles in providing safe abortion care and post-abortion contraception	Health systems	Published 2015
10	Guía de la OMS sobre políticas nacionales de precios de medicamentos	WHO guideline on country pharmaceutical pricing policies	Health systems	Published 2013
11	Optimización de los roles de los trabajadores de salud para la salud materna y neonatal	Optimizing health worker roles for maternal and newborn health	Health systems	Published 2012
12	Kit de herramientas de Centros de Atención Primaria de Salud (PHC) para personas mayores	Age-friendly Primary Health Care (PHC) Centres Toolkit	Health systems	Published 2007
13	Mejores prácticas para inyecciones y procedimientos relacionados con el kit de herramientas	Best practices for injections and related procedures toolkit	Health systems	Published 2010
14	Aumentar el acceso a los trabajadores de salud en áreas remotas y rurales a través de una mejor retención	Increasing access to health workers in remote and rural areas through improved retention	Health systems	Published 2010
15	Guía de Práctica Clínica ​(GPC): Niños, niñas con diagnóstico de asma	Clinical Practice Guideline: Children diagnosed with asthma	Paediatric pneumology	Published 2013
16	Guía de Práctica Clínica ​(GPC): Recién nacido sano	Clinical Practice Guideline: Healthy newborn	Neonatology	Published 2013
17	Guía de Práctica Clínica ​(GPC): Detección de anomalías congénitas en el recién nacido	Clinical Practice Guideline: Detection of congenital anomalies in the newborn	Neonatology	Published 2013
18	Guía de Práctica Clínica ​(GPC) del recién nacido prematuro	Clinical Practice Guideline of the premature newborn	Neonatology	Updated 2014
19	Guía de Práctica Clínica ​(GPC): Recién nacido con trastorno respiratorio	Clinical Practice Guideline: Newborn with respiratory disorder	Neonatology	Published 2013
20	Guía de Práctica Clínica ​(GPC): Recién nacido con sepsis neonatal temprana	Clinical Practice Guideline: Newborn with early neonatal sepsis	Neonatology	Published 2013
21	Guía de Práctica Clínica ​(GPC): Recién nacido con asfixia perinatal	Clinical Practice Guideline: Newborn with perinatal asphyxia	Neonatology	Published 2013
22	Guía de Práctica Clínica ​(GPC): Enfermedad diarreica aguda en niños menores de 5 años	Clinical Practice Guideline: Acute diarrheal disease in children under 5 years of age	General paediatrics	Published 2013
23	Guía de Práctica Clínica ​(GPC): Leucemia linfoide aguda y leucemia mieloide aguda en niños, niñas y adolescentes	Clinical Practice Guideline: Acute lymphoid leukemia and acute myeloid leukemia in children and adolescents	Paediatric oncology	Published 2013
24	Guía de Práctica Clínica ​(GPC): Linfoma de Hodgkin y Linfoma no Hodgkin en niños, niñas y adolescentes	Clinical Practice Guideline: Hodgkin lymphoma and non-Hodgkin lymphoma in children and adolescents	Paediatric oncology	Published 2013
25	Guía de Práctica Clínica ​(GPC): Complicaciones del embarazo, parto, o puerperio	Clinical Practice Guideline: Complications of pregnancy, delivery, or puerperium	Gynaecology and obstetrics	Published 2013
26	Guía de Práctica Clínica ​(GPC): Infecciones de trasmisión sexual y otras infecciones del tracto genital	Clinical Practice Guideline: Sexually transmitted infections and other genital tract infections	Gynaecology and obstetrics	Published 2013
27	Guía de Práctica Clínica ​(GPC): Síndrome coronario agudo	Clinical Practice Guideline: Acute coronary syndrome	Internal medicine	Updated 2017
28	Guía de Práctica Clínica ​(GPC): Hipertensión Arterial Primaria	Clinical Practice Guideline: Primary hypertension	Internal medicine	Updated 2017
29	Guía de Práctica Clínica ​(GPC): Cáncer de mama	Clinical Practice Guideline: Breast cancer	Oncology	Updated 2017
30	Guía de Práctica Clínica ​(GPC): Cáncer de colon y recto	Clinical Practice Guideline: Colon and rectal cancer	Oncology	Updated 2017
31	Guía de Práctica Clínica ​(GPC): Cáncer de próstata	Clinical Practice Guideline: Prostate cancer	Oncology	Published 2013
32	Guía de Práctica Clínica ​(GPC): Episodio depresivo o trastorno depresivo recurrente	Clinical Practice Guideline: Depressive episode or recurrent depressive disorder	Psychiatry and mental health	Published 2013
33	Guía de Práctica Clínica ​(GPC): Abuso o dependencia del alcohol	Clinical Practice Guideline: Alcohol abuse or dependence	Psychiatry and mental health	Published 2013
34	Guía de Práctica Clínica para la prevención, detección temprana y manejo inicial de alteraciones del crecimiento y neurodesarrollo en menores de 10 años	Clinical Practice Guideline for the prevention, early detection and initial management of growth and neurodevelopmental disorders in children under 10 years of age	General paediatrics	Published 2015
35	Guía de Práctica Clínica para la detección temprana, tratamiento, seguimiento y rehabilitación de pacientes con artritis idiopática juvenil	Clinical Practice Guideline for the early detection, treatment, follow-up and rehabilitation of patients with juvenile idiopathic arthritis	Paediatric rheumatology	Published 2014
36	Guía de Práctica Clínica para la detección temprana, tratamiento, seguimiento y rehabilitación de pacientes con artritis reumatoide	Clinical Practice Guideline for the early detection, treatment, follow-up and rehabilitation of patients with rheumatoid arthritis	Rheumatology	Published 2014
37	Guía de Práctica Clínica para la prevención y detección temprana, tratamiento y seguimiento de las dislipidemias en población mayor de 18 años (1)	Clinical Practice Guideline for the prevention and early detection, treatment and follow-up of dyslipidemias in a population over 18 years of age	Internal medicine	Published 2014
38	Guía de Práctica Clínica para la prevención, diagnóstico, tratamiento, rehabilitación y seguimiento de la enfermedad pulmonar obstructiva crónica (EPOC)	Clinical Practice Guideline for the prevention, diagnosis, treatment, rehabilitation and follow-up of chronic obstructive pulmonary disease (COPD)	Pneumology	Published 2014
39	Guía de Práctica Clínica para el diagnóstico, tratamiento y rehabilitación funcional de la esquizofrenia	Clinical Practice Guideline for diagnosis, treatment and functional rehabilitation of schizophrenia	Psychiatry	Published 2014
40	Guía de Práctica Clínica para el diagnóstico, tratamiento y rehabilitación de pacientes con trauma craneoencefálico	Clinical Practice Guideline for the diagnosis, treatment and rehabilitation of patients with cranioencephalic trauma	Neurology	Published 2014
41	Guía de Práctica Clínica para el diagnóstico, tratamiento y seguimiento del cáncer de piel: queratosis actínica	Clinical Practice Guideline for the diagnosis, treatment and monitoring of skin cancer: actinic keratosis	Oncology	Published 2014
42	Guía de Práctica Clínica para el diagnóstico, tratamiento y seguimiento del cáncer de piel: cáncer escamo celular	Clinical Practice Guideline for diagnosis, treatment and follow-up of skin cancer: cell-scale cancer	Oncology	Published 2014
43	Guía de Práctica Clínica para el diagnóstico, tratamiento y seguimiento del cáncer de piel: cáncer basocelular	Clinical Practice Guideline for the diagnosis, treatment and monitoring of skin cancer: basal cell cancer	Oncology	Published 2014
44	Guía de Práctica Clínica para la detección, tratamiento y seguimiento de leucemias linfoide y mieloide en población mayor de 18 años	Clinical Practice Guideline for the detection, treatment and follow-up of lymphoid and myeloid leukemias in a population over 18 years of age	Oncology	Published 2018
45	Guía de Práctica Clínica para la detección, tratamiento y seguimiento de linfomas Hodgkin y no Hodgkin en población mayor de 18 años	Clinical Practice Guideline for the detection, treatment and follow-up of Hodgkin and non-Hodgkin lymphomas in a population over 18 years of age	Oncology	Published 2018
46	Guía de Práctica Clínica para la prevención, detección, tratamiento y rehabilitación del cáncer de pulmón	Clinical Practice Guideline for the prevention, detection, treatment and rehabilitation of lung cancer	Oncology	Published 2016
47	Guía de Práctica Clínica para la detección temprana, atención integral, seguimiento y rehabilitación de pacientes con diagnóstico de distrofia muscular de Duchenne, Becker y distrofia miotónica	Clinical Practice Guideline for early detection, comprehensive care, follow-up and rehabilitation of patients diagnosed with Duchenne muscular dystrophy, Becker and myotonic dystrophy	Physical medicine and rehabilitation	Published 2014
48	Guía de Práctica Clínica para la prevención, diagnóstico tratamiento y rehabilitación de fibrosis quística	Clinical Practice Guideline for the prevention, diagnosis, treatment and rehabilitation of cystic fibrosis	Pneumology	Published 2014
49	Guía de Práctica Clínica basada en la evidencia científica para la atención de la infección por VIH/SIDA en adolescentes (mayores de 13 años de edad) y adultos	Clinical Practice guideline based on scientific evidence for the care of HIV/AIDS infection in adolescents (over 13 years of age) and adults	Infectiology	Published 2014
50	Guía de Práctica Clínica basada en la evidencia científica para la atención de la infección por VIH en niñas y niños menores de 13 años	Clinical Practice Guideline based on scientific evidence for the care of HIV infection in girls and boys under 13 years	Infectiology	Published 2014
51	Guía de Práctica Clínica para la atención integral de la sífilis gestacional y congénita	Clinical Practice Guideline for comprehensive care of gestational and congenital syphilis	Infectiology	Published 2014
52	Guía de Práctica Clínica para la evaluación del riesgo y manejo inicial de la neumonía en niños y niñas menores de 5 años y bronquiolitis en niños y niñas menores de 2 años	Clinical Practice Guideline for risk assessment and initial management of pneumonia in children under 5 years of age and bronchiolitis in children under 2 years of age	Paediatric pulmonology	Published 2014
53	Guía de Práctica Clínica para la identificación y el manejo clínico de la tos ferina en menores de 18 años de edad (actualización 2014)	Clinical Practice Guideline for the identification and clinical management of pertussis in children under 18 years of age (2014 update)	Paediatric infectiology	Published 2014
54	Guía de Práctica Clínica para la detección y manejo de lesiones precancerosas de cuello uterino	Clinical Practice Guideline for the detection and management of precancerous lesions of cervix	Oncology	Published 2016
55	Guía de Práctica Clínica para el manejo del cáncer de cuello uterino invasivo	Clinical Practice Guideline for the management of invasive cancer of cervix	Oncology	Published 2016
56	Guía de Práctica Clínica para diagnóstico y manejo de epilepsias en adultos y niños, en la atención primaria y secundaria	Clinical Practice Guideline for diagnosis and management of epilepsies in adults and children, in primary and secondary care	Neurology	Published 2014
57	Guía de Práctica Clínica en prevención, detección temprana,diagnóstico y tratamiento de defecto refractivos en menores de 18 años	Clinical Practice Guideline: on prevention, early detection, diagnosis and treatment of refractive errors in children under 18 years of age	Ophthalmology and optometry	Published 2016
58	Guía de Práctica Clínica en prevención, detección temprana,diagnóstico y tratamiento de ambliopía en menores de 18 años	Clinical Practice Guideline: on prevention, early detection, diagnosis and treatment of amblyopia in children under 18 years	Ophthalmology and optometry	Published 2016
59	Guía de Práctica Clínica para el diagnóstico, tratamiento y seguimiento de diabetes gestacional	Clinical Practice Guideline for diagnosis, treatment and follow-up of gestational diabetes	Endocrinology and gynaecology	Published 2016
60	Guía de Práctica Clínica para el diagnóstico, tratamiento y seguimiento de diabetes tipo I	Clinical Practice Guideline for diagnosis, treatment and follow-up of type I diabetes	Endocrinology	Published 2016
61	Guía de Práctica Clínica para el diagnóstico, tratamiento y seguimiento de diabetes tipo II en población mayor de 18 años	Clinical Practice Guideline for the diagnosis, treatment and follow-up of type II diabetes in a population over 18 years of age	Endocrinology	Published 2016
62	Guía Práctica Clínica para la prevención, diagnóstico, tratamiento de la obesidad en adultos	Clinical Practice Guideline for the prevention, diagnosis, treatment of obesity in adults	Internal medicine and endocrinology	Published 2016
63	Guía de Práctica Clínica para la prevención, diagnóstico, tratamiento y rehabilitación de la falla cardiaca en población mayor de 18 años	Clinical Practice Guideline for the prevention, diagnosis, treatment and rehabilitation of heart failure in a population over 18 years of age	Internal medicine and cardiology	Published 2016
64	Guía de Práctica Clínica de diagnóstico, tratamiento y rehabilitación del episodio agudo de los accidentes cerebro-vasculares isquémico en población mayor de 18 años	Clinical Practice Guideline for diagnosis, treatment and rehabilitation of acute episode of ischemic stroke in a population over 18 years of age	Neurology	Published 2016
65	Guía de Práctica Clínica para el diagnóstico y tratamiento preoperatorio, intraoperatorio y postoperatorio de la persona amputada, la prescripción de la prótesis y la rehabilitación integral	Clinical Practice Guideline for the diagnosis and preoperative, intraoperative and postoperative treatment of the amputee, the prescription of the prosthesis and the integral rehabilitation	Physical medicine and rehabilitation	Published 2016
66	Guía de Práctica Clínica sobre diagnóstico y tratamiento de hepatitis B crónica​	Clinical Practice Guideline on diagnosis and treatment of chronic hepatitis B	Infectiology	Published 2016
67	Guía de Práctica Clínica para la tamización, diagnóstico y tratamiento de personas con infección por el virus de la hepatitis C​	Clinical Practice Guideline for screening, diagnosis and treatment of people with hepatitis C virus infection	Infectiology	Published 2016
68	​Guía de Práctica Clínica para cuidados paliativos (adopción)	Clinical Practice Guideline for palliative care (adoption)	Palliative care	Published 2017
69	Guía de Práctica Clínica: Enfermedad renal crónica	Clinical Practice Guideline: Chronic kidney disease	Nephrology	Published 2017
70	Guía de Práctica Clínica para la prevención, diagnóstico y tratamiento de la ideación conducta suicida (adopción)	Clinical Practice Guideline for the prevention, diagnosis and treatment of suicidal ideation (adoption)	Psychiatry	Published 2018
71	Guía de Práctica Clínica para el diagnóstico y tratamiento del trastorno neurocognoscitivo mayor (demencia) (adopción)​	Clinical Practice Guideline for the diagnosis and treatment of major neurocognitive disorder (dementia) (adoption)	Psychiatry	Published 2018
72	Guía de Práctica Clínica para el uso de componentes sanguíneos​	Clinical Practice Guideline for the use of blood components	Haematology	Published 2018

Health systems guidelines (HSGs) are relatively new tools compared with CPGs. HSGs provide systematically developed recommendations focused on addressing challenges regarding health system arrangements, including financial (e.g. financing strategies and funding mechanisms), delivery (e.g. who provides healthcare, where, how and with what support), governance (e.g. professional, organizational or commercial authority) and implementation strategies required to provide appropriate programs and services to those who need them [13]. One of the approaches taken by the WHO to strengthen health systems has been to develop health systems guidance at the global level. Global guidance has three general purposes: first, pooling resources and knowledge to recommend possible solutions to problems faced by different countries; second, serving as a guidance to be used in the development of national guidelines; and third, informing the development of policy at the national or subnational level. In all cases, for a guidance to have an impact, recommendations need to be adapted to a particular context or setting, whether national or subnational [14].

The overwhelming number of recommendations, lack of implementation experience and barriers that commonly emerged at the moment of implementation [15–17] left a considerable proportion of the recommendations unimplemented. Several of these recommendations focus on changing clinical practice and the behaviours of healthcare providers, patients and citizens. However, one of the barriers that we have identified in our previous research and participation in the development of these guidelines is the necessity of making changes to the health system arrangements [18–20].

We identified that some recommendations require changes in the governance arrangements of the Colombian health system, for instance, a change in the scope of practice of health professionals (e.g. general practitioners allowed to formulate medications exclusively managed by specialists) or a modification in the commercial authority to import or approve some technologies in the national territory (e.g. authorizing the use of zinc for diarrhoea in children). Other recommendations imply a change in financial arrangements, for example, the inclusion of technologies/services in the benefits plan (e.g. including a new statin in the benefits plan), the allocation of financial resources to purchase some products or services not previously funded, or modification in the mechanisms for remunerating providers. Other recommendations need a change in the delivery arrangements, among them, designing new strategies to meet consumer’s needs or establishing where the care is provided.

Considering this context, our research question was: What prioritized recommendations are pending implementation in Colombia, and which of them need changes in Colombian health system arrangements? We looked at recommendations made in 58 national CPGs and 14 WHO HSGs. As a result of the Colombian government’s commitment to receive advice from the WHO, we included the WHO HSGs in this review. Additionally, many of the WHO HSG recommendations are directed to make health system changes that improve the performance of the Colombian health sector; therefore, in our rationale, recommendations from WHO are priorities for the Colombian health system.

This study is part of a larger research program designed to promote evidence-informed decision making for implementing recommendations. Identifying key recommendations is the first step in prioritizing them and in recognizing health system challenges that must be addressed during implementation.

## Methods

This was a document analysis of all 58 CPGs from the Colombian Ministry of Health and all 14 HSGs from the World Health Organization that were identified by a search between December 2019 and April 2020 (Table 1).

### Document retrieval

The research team searched the CMoH web repository which contained all of the national guidelines to identify and retrieve the most updated versions of the Colombian CPGs (since 2021, this repository is no longer available). To identify WHO HSGs, we searched in the official catalogue of health systems guidance documents [21]. Searches were carried out independently by two members of the research team between December 2019 and April 2020.

We did not exclude any documents; however, we included the most recent version when guidelines had been updated.

### Criteria for inclusion of recommendations

The unit of analysis in this study was the guideline recommendation. According to our objective of identifying priority recommendations for implementation, we selected all the recommendations that were classified by the development groups of each CPG as “key recommendation”, “priority recommendation” or “tracer recommendation” (i.e. recommendations prioritized given their most significant potential impact on patients’ outcomes/experiences, or over the health system). When the CPG did not provide a distinction for recommendations prioritized, we contacted the leader of the CPG development groups to request assistance on defining those recommendations prioritized. We included all the recommendations in the HSGs from WHO, as we considered supranational guidance to contain priority recommendations.

### Data extraction

A template was developed to systematically extract information about two domains that characterize both guidelines and recommendations. The first domain was the CPG/HSG characteristics (i.e. name, year of publication, institution that created the document, classification of the guideline according to field or medical speciality). The second domain was related to the characteristics of the recommendations, which included a complete description of the recommendation, its strength (i.e. using grades A–D, or classified according to the Grading of Recommendations, Assessment, Development and Evaluation (GRADE) system), and direction (i.e. in favour or against) as determined by the CPG/HSG development groups [22]. Recommendation graded A, B or strong were considered strong, and those graded C, D and weak were considered weak, other recommendations were classified as conditional or recommended under specific circumstances [20]. We also recorded the type of intervention or service addressed by the recommendation (e.g. screening, diagnostic test or service, medication, device, procedure, service, education/counselling). We defined the update status of the recommendation (i.e. classified as updated if it was published/updated in the last 4 years, 2016–2020; close to outdated if published 5 years ago, in 2015; and outdated if the article was published 6 or more years ago). This information was included because it will be useful to policy-makers prioritizing recommendations that need to be updated. We performed a pilot of the data extraction procedure with 50 recommendations to complete a process of calibration among the research team. All the data extraction was performed independently by two researchers and checked by the principal investigator. Discrepancies were solved by consensus or by a third party.

After completing the dataset with all the recommendations prioritized, we incorporated one additional dimension that captured the identification and analysis of health system requirements for recommendation implementation. First, when a recommendation addressed a medication or device, we searched in two government databases to identify whether the medication/device was approved in Colombia for use as indicated by the recommendation [23] and if it was publicly funded [24]. With WHO recommendations, two researchers with experience of the healthcare system (P.V. and C.M.V.), independently and by duplicate, assessed whether the recommendation was implemented completely, partially or not at all in the Colombian health system. The criteria to decide the level of implementation of the recommendations was based on reviews of the plans and programs in place in the country. The discrepancies in the assessment of the level of implementation were solved by consensus between reviewers.

### Data analysis

For all the recommendations included, two researchers (P.V. and C.M.V.) independently assessed whether any change in the health system was necessary for implementing the recommendation and what type of change was required. This assessment was checked by three other research team members (J.S.F., L.H.L., C.Y.V. and V.M.V.), and discrepancies were solved by consensus in a meeting.

### Effective Practice and Organisation of Care (EPOC) taxonomy

Using the EPOC taxonomy, explained further below, two researchers (C.M.V. and P.V.) classified the changes required in the categories of governance, financial and delivery arrangements, and selected the appropriate type according to the options available in this comprehensive taxonomy. This classification was checked by other research team members (J.S.F., L.H.L., C.Y.V. and V.M.V.). In a final round of discussion, the whole team consistently decided, by consensus, the type of changes to Colombian health system arrangements necessary for implementing each recommendation.

The EPOC taxonomy was initially developed as a checklist to assist in the inclusion assessment of studies in systematic reviews [25]. However, the EPOC taxonomy has also been used to classify factors involved in implementation by distinguishing them across four domains, each with categories and subcategories. Those four domains are delivery arrangements, financial arrangements, governance arrangements and implementation strategies interventions [26]. The delivery arrangements dimension includes how and when care is delivered; where care is provided and changes to the healthcare environment; who provides care and how the healthcare workforce is managed; coordination of care and management of care processes; and information and communication technology (ICT).

The financial arrangements dimension considers changes in how funds are collected, insurance schemes, mechanisms for the payment of health services, and the use of targeted financial incentives for health professionals and healthcare organizations. The governance arrangements dimension addresses rules or processes that affect how powers are exercised, particularly about authority and accountability for health policies, organizations, commercial products and health professionals. The dimension of implementation strategies interventions includes factors related to healthcare organizations, the behaviour of healthcare professionals and the use of health services by healthcare recipients.

In this study, we used the dimensions of delivery, financial and governance arrangements because they help identify changes at the health system level. However, the dimension of implementation strategies interventions was not used given that this dimension addressed changes and interventions at the individual or institutional level, which is not the focus of this research.

We used descriptive statistical analysis of absolute and relative frequencies. Analysis was performed using SPSS 21.

### Development of an interactive tool

The research team decided to develop an interactive tool that presents the database created in this project. As the database was gaining complexity, the research team understood the relevance of providing an interactive tool that allows stakeholders to navigate and identify what recommendations are prioritized, according to different variables, and recognize what changes in health system arrangements are necessary for implementation. We linked our different datasets that contain guidelines, recommendations and all the extracted data previously mentioned and built an interface that connects the information. We developed this tool, which is publicly available (see Availability of data and materials) [27]. This tool is currently published in Spanish, as the purpose was to inform policy-makers and stakeholders in Colombia.

## Results

**Fig. 1:**
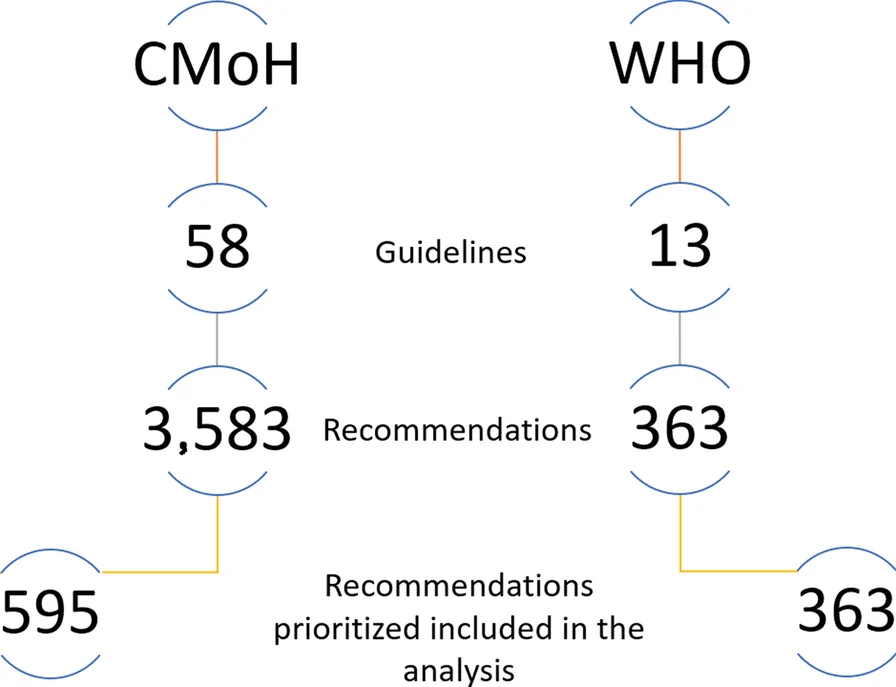
Flowchart of recommendations included in the analysis

We identified 3583 recommendations in 58 Colombian CPGs and 363 recommendations in the group of 14 WHO HSGs. A total of 958 recommendations were included in this analysis, of which 595 were recommendations prioritized in the Colombian CPGs, and 363 were from the WHO HSGs (Fig. 1). Most of the Colombian CPGs (*n* = 56) had explicitly identified key/priority recommendations; for two guidelines, we contacted the leader of the development group to obtain a list of priority recommendations.

### General characteristics of recommendations

Among all recommendations included, 393 were updated (41.0%), 425 were outdated (44.4%) and 140 were close to being outdated (14.6%) (Table 2). Recommendations covered the following fields or medical specialities: maternal and child health (*n* = 223); chronic nontransmissible diseases (*n* = 171); cancer care (*n* = 150); sexual and reproductive health (*n* = 142); infectious diseases (*n* = 68); patient safety (*n* = 46); mental health (*n* = 39); orphan or rare diseases (*n* = 31); pharmaceutical and health system policies (*n* = 28); oral, visual and hearing health (*n* = 25); community-level interventions for elderly population (*n* = 13); conditions associated with trauma (*n* = 12); and digital interventions (*n* = 10).

**Table 2:** Update status of the recommendations

Status	CMoH (*n* = 595)	WHO (*n* = 363)	Total (*n* = 958)
Outdated	320 (53.8%)	105 (28.9%)	425 (44.4%)
Updated	257 (43.2%)	136 (37.5%)	393 (41.0%)
Close to outdated	18 (3.0%)	122 (33.6%)	140 (14.6%)

Regarding the strength and direction of the recommendations, 540 were strong (56.4%), 189 were weak (19.7%), 173 were suggested or recommended in a specific context (18.1%) and 37 were recommendations of good practice or based on experts’ opinions (3.9%). For 19 recommendations, it was not possible to identify their strength (2%). Most of the recommendations were in favour of the intervention (*n* = 861, 89.9%), and only 97 were against the intervention (10.1%) (Table 3).

**Table 3:** Strength and direction of the recommendations by publisher

	CMoH		WHO		Total	
	In favour	Against	In favour	Against	In favour	Against
Strong/recommended	342 (62.1%)	31 (70.5%)	121 (39.0%)	46 (86.8%)	463 (53.8%)	77 (79.4%)
Weak/suggested	159 (28.9%)	11 (25.0%)	17 (5.5%)	2 (3.8%)	176 (20.4%)	13 (13.4%)
Recommended in specific context/within the context of rigorous research	0	0	59 (19.0%)	0	59 (6.9%)	0
Suggested: conditional/within the context of rigorous research/with targeted monitoring and evaluation	2 (0.36%)	0	107 (34.5%)	5 (9.4%)	109 (12.7%)	5 (5.2%)
Good practice point	11 (2.0%)	1 (2.3%)	6 (1.9%)	0	17 (2.0%)	1 (1.0%)
Based on expert’s opinion	19 (3.4%)	0	0	0	19 (2.2%)	0
No information	18 (3.3%)	1 (2.3%)	0	0	18 (2.1%)	1 (1.0%)
Total	551	44	310	53	861	97

The most common types of technologies addressed by the recommendations were medications (*n* = 228); therapeutic procedures (*n* = 155); diagnosis tests or procedures (*n* = 119); and counselling and education (*n* = 138) (Table 4).

**Table 4:** Interventions addressed in the recommendations

Intervention	CMoH		WHO		All recommendations	
	Total (*n* = 595)	Strong *n* (%^a^)	Total (*n* = 363)	Strong *n* (%^a^)	Total (*n* = 958)	Strong *n* (%^a^)
Health system intervention	0		55	17 (4.7%)	55	17 (3.9%)
Clinical practice	142	57 (9.6%)	43	25 (6.9%)	185	82 (18.6%)
Service	0		9	5 (1.4%)	9	9 (2.0%)
Procedure	46	17 (2.9%)	109	66 (18.2%)	155	83 (18.9%)
Drug	143	71 (11.9%)	85	25 (6.9%)	228	96 (21.8%)
Device	29	12 (2%)	9	1 (0.3%)	38	13 (3.0%)
Surgery	0		16	8 (2.2%)	16	8 (1.8%)
Self-management	0		10	4 (1.1%)	10	4 (0.9%)
Counselling, education and related activities	116	31 (3.4%)	22	12 (3.3%)	138	43 (9.8%)
ICT services and related activities	0		5	1 (0.3%)	5	1 (0.2%)
Screening/diagnosis test	119	84 (14.1%)	0		119	84 (19.1%)
Total		272 (45.7%)		164 (45.2%)		440 (45.9%)

^a^Relative frequency using as denominator the total number of recommendations in each category

*ICT* information and communication technologies

### Changes necessary in health system arrangements

Among the 363 recommendations from the WHO HSGs, 244 (67.2%) were not implemented in Colombia. From the total of 958 recommendations, 518 (54.1%) required a change in health system arrangements for implementation (Table 5).

**Table 5:** Health system arrangement required for implementing the recommendations

Arrangement	CMoH	WHO	Total
Need a health system arrangement^a^	260	258	518
Governance arrangement			
Accreditation	22 (31.9%)	9 (4.7%)	31 (12.0%)
Decision-making authority about who is covered and what can or must be provided to them	19 (27.5%)	3 (1.6%)	22 (8.5%)
Stewardship of the non-state sector’s role in financing and delivering	12 (17.4%)	21 (11.1%)	33 (12.7%)
Training and licensure requirements and scope of practice	4 (5.8%)	155 (81.6%)	159 (61.4%)
Other governance arrangements^b^	12 (17.4%)	2 (1.1%)	14 (5.4%)
Subtotal	69	190	259
Delivery arrangement			
List of covered/reimbursed organizations, providers, services and products	80 (100%)	12 (54.5%)	92 (90.2%)
Other financial arrangements^c^	0	10 (45.5%)	10 (9.8%)
Subtotal	80	22	102
Delivery arrangement			
Availability of care	174 (69.6%)	217 (85.8%)	391 (77.7%)
Timely access	51 (20.4%)	63 (24.9%)	114 (22.7%)
Staff training	39 (15.6%)	170 (67.2%)	209 (41.6%)
Role expansion and extension	2 (0.8%)	153 (60.5%)	155 (30.8%)
Culturally appropriate care	12 (4.8%)	58 (22.9%)	70 (13.9%)
Other delivery arrangements^d^	67 (26.8%)	45 (17.8%)	80 (15.9%)
Subtotal	250	253	503

^a^Recommendations might require more than one type of health system arrangement

^b^Sales and dispensing; accountability of the state sector’s role in financing and delivering; licensure and registration requirements; networks/multi-institutional arrangement

^c^Funding organizations, remunerating providers, included in benefits plan

^d^Continuity of care; staff/self shared decision-making; skill mix – communication and case discussion between distant health professionals, quality and safety; other ICT that supports individuals who provide care; other ICT that supports individuals who receive care; quality monitoring and improvement systems; staff workload/workflow/intensity; physical structure, facilities and equipment; system – need, demand and supply, electronic health record; case management

Changes in governance arrangements were necessary to implement 259 (27%) recommendations. Principally, we identified changes necessary in policies of training and licensure requirements, plus changes to the scope of practice (*n* = 159, 61.4% of all recommendations that need a governance arrangement). We identified 89 recommendations that required completion of the licensure and registration requirements of the Colombian regulatory agency (INVIMA) to be authorized and used as the recommendation indicates. A total of 22 recommendations required a change in the decision-making authority about who is covered by public health insurance and what can or must be provided.

We assessed that the implementation of 46 recommendations goes beyond the scope of policy decision-making of the CMoH. These 46 recommendations were in three HSGs of the WHO. Two of these HSGs focused on health worker roles in providing safe abortion care, post-abortion contraception, and self-care interventions for sexual health and reproductive health rights. The other guideline was focused on national pharmaceutical pricing policies. We also identified that some recommendations required changes related to the process of accreditation, stewardship of the non-state sector’s role in financing and delivering healthcare services, and accountability of the state sector’s role in financing and delivering.

Changes in financial arrangements were identified as necessary in 102 (10.6%) recommendations, principally related to changes to the list of covered/reimbursed organizations, providers, services and products (*n* = 92, 90.2% of all recommendation that need a financial arrangement). We identified 86 recommendations that required the intervention to be included in the benefits plan or to clarify and communicate that the intervention is publicly funded; these recommendations covered medications (*n* = 58); devices and prosthesis (*n* = 6); diagnostic tests (*n* = 14); education and counselling (*n* = 3); procedures (*n* = 2); and other kinds of services (*n* = 3). Almost 65% of recommendations that required the inclusion of a service/technology in the benefits plan were graded by the CPG development group as strong/grade A–B.

Lastly, 503 recommendations (52.5%) required a change in delivery arrangements; the 4 most common were (1) availability of care (*n* = 391), (2) staff training (*n* = 209), (3) role expansion and extension (*n* = 155), and (4) timely access (*n* = 114) (Table 3). We identified 70 recommendations that required a change in the delivery arrangements to guarantee that it will be culturally appropriate; 56 of those recommendations were for sexual and reproductive health. For instance, most of these recommendations came from the WHO HSG “Health worker roles in providing safe abortion care and post-abortion contraception”; even if abortion were not illegal in Colombia, many changes in the delivery would be necessary to guarantee that it will be culturally appropriate.

The three Colombian CPGs that required the most changes in health system arrangements were (1) sexually transmitted disease (35 changes), (2) heart failure (21 changes) and (3) juvenile idiopathic arthritis (18 changes). The top three for the WHO HSGs were (1) health worker roles to key maternal services (168 changes), (2) health worker roles in abortion (140 changes) and (3) health policy to optimize community health worker programmes (34 changes).

## Discussion

We described a process to identify and classify CPG/HSG recommendations prioritized for implementation. From a set of 3583 recommendations in the Colombian CPGs, we assessed the requirement of change in health system arrangements of 595 high-priority recommendations. We also evaluated 363 recommendations of World Health Organization HSGs.

The development of the Colombian CPGs required a significant investment of resources, and it is alarming that at the time of writing this paper, 338 (56.8%) priority recommendations are outdated. This proportion of outdated recommendations was slightly higher in the set of 14 WHO HSGs assessed in this research (*n* = 227, 62.5%). This is an opportunity to prioritize the allocation of resources to update some of those recommendations.

Of note, 90% of the recommendations favoured a technology/service, which indicates that something new should be done in the medical practice or included in the health system. However, this finding also shows that 97 recommendations require a behaviour change and education to cease practices that do not benefit the patient or could cause harm, as indicated by the research evidence. In 10 of these recommendations, we identified that a change in the governance arrangements was necessary to enforce compliance with the recommendation. For example, we identified that tanning chambers were recommended for discontinuation. This requires the commitment of health authorities to educate the population about the risks and design regulatory mechanisms to prohibit their sale.

We identified 518 recommendations that require a change in Colombian health system arrangements. The most common change was related to the availability of care. Many of the recommendations refer to approved and publicly funded technologies/services that are not widely available or accessible to different population groups. For instance, some diagnosis tests are publicly funded but are only available in capital cities with strong healthcare infrastructure, or the same test is provided by one insurance company and not by another. Other common changes identified refer to inclusion in the benefits plan; 67.4% of these recommendations were about medications, and most (63.8%) were graded as strong/grade A–B. This provides an opportunity to check these recommendations and prioritize cost-effectiveness analysis that will inform the policy decision-making about publicly funding those medications.

WHO recommendations represented 49.8% of those recommendations that required a health system change. In 46 of these WHO recommendations, we assessed that their implementation goes beyond the scope of policy decision-making of the CMoH. Those recommendations focused on health workers’ roles in providing safe abortion care, post-abortion contraception and self-care interventions for sexual health and reproductive health rights. Some of the recommendations in this guidance require that abortion be legal in Colombia, which is beyond the scope of decision-making by the CMoH and depends on the approval of a law in this matter. The other guideline focuses on national pharmaceutical pricing policies. Some recommendations require changes in the Ministry of Foreign Trade; in other cases, decisions in the CMoH are constrained by the bilateral free trade agreement with the United States.

Regarding barriers for implementing recommendations from CPGs/HSGs, research carried out in Colombia found that governance, financial and delivery arrangements of the Colombian health system are determining factors in implementing CPGs. For instance, financial arrangements between insurance companies and health provider institutions were identified as barriers to the implementation of recommendations related to the continuity and opportunity of care for amputee patients [28]. Similar barriers were identified in a meta-review about individual, health system, and contextual barriers and facilitators for the implementation of CPG in low- and middle-income countries. In this review, authors identified barriers such as lack of access to medicines; deficiencies in transport and communication systems; fear and confusion among the implementers, policy-makers and government; unequal distribution of resources; poverty; problems in the care of minorities; and costs of care [29].

Lynch et al. recently presented a feasible strategy to prioritize recommendations for implementation considering the preferences of both consumers and health professionals [6]. The authors found that the most relevant criteria for consumers and health professionals to prioritize a recommendation for implementation were how compelling and robust the evidence supporting the recommendation is, with a preference for strong recommendations [6]. In our study, we also presented the recommendations according to their strength as a way to inform policy-makers and stakeholders that those recommendations are strongly supported by evidence or come from a robust process of consensus among the guideline development group.

### Strengths and limitations

We identified previous studies in Colombia that addressed the implementation of specific CPGs [30, 31], and studies that sampled CPGs in different countries and assessed their quality and the presence of some elements, such as a plan for implementation [32, 28]. Previous reviews lack the comprehensiveness to include all guidelines and recommendations available for Colombia; those reviews omitted to consider what changes are necessary to implement prioritized recommendations. The novelty of our review is that it systematically consolidates all the CPG/HSG-prioritized recommendations for the Colombian health system. We developed this study on the basis of the foundations of rigorous work performed by different guideline development groups. We used what they considered to be prioritized recommendations, how they graded the recommendation’s strength and direction, and how they classified the scope and focus of the guideline. When analysing the necessary changes to the Colombian health system arrangements, we selected a consistent approach through the internationally acceptable EPOC taxonomy. We had different rounds of discussion until we agreed on the final assessment. Given that this information might be presented in different ways that make sense for policy-makers and stakeholders interested in or advocating for their implementation, we developed a publicly available tool that presents the database of prioritized recommendations [27]. In a future article, we plan to describe how to move from the recommendations to an actual case of prioritization to plan the implementation.

As our methodology is based on the strengths of the development process of the CPGs and HSGs, this is also a source of our study’s limitations. One significant limitation is that not all guidelines were developed with the same methodology; some used GRADE, while others did not. Older Colombian CPGs used various methods and graded the recommendations using different systems. Old WHO HSGs were also developed using a non-GRADE methodology. When we consolidated all the recommendations and classified them by strength and direction, there was no easy way to harmonize these differences. Therefore, we presented the strong recommendations and A as belonging to the same group, but these systems do not speak about the same things. GRADE assessed the recommendations on the basis of the researchers’ certainty in the evidence found and the likelihood that all persons would choose that intervention considering the certainty on that evidence [22]. The system of the American College of Cardiology and the American Heart Association (ACC/AHA) Task Force uses the letters A, B and C to indicate the quality of evidence which are then divided into levels I, II and III that indicate how sure the group is about the effectiveness of the technology/service considered [34]. We consider that further research might consider systems for harmonizing different models of grading the strengths of recommendations. An additional limitation is the lack of appraisal of the guidelines included. We could have applied AGREE II to this assessment; however, we decided that our unit of analysis is the recommendation and not the guideline as a whole, and that the quality of the guideline does not necessarily correlate with the quality of the recommendation.

## Conclusions

The implications for practice that we expect of this research are the utilization of this dataset to identify recommendations for the process of implementation. In a healthcare environment with multiple competing demands for finite resources, potential service improvements need to be prioritized from a large set of evidence-based recommendations. Policy-makers and stakeholders might shortlist the recommendations that are up for implementation by selecting the conditions, age groups and relevance of the diseases according to different variables and move to identify what is necessary to initiate the process of implementation. The dataset and tool developed for consulting the recommendations can help to identify recommendations that require updates and to prioritize the research process for updating the evidence. The implication for policy includes that the CMoH resumes the updating of the CPGs while developing plans of implementation for the recommendations prioritized. Political will and commitment to the changes to the governance arrangements are key to initiating this process.

The next step in the research is exploring how this database can support selecting recommendations and planning their implementation in real life. As Lynch et al. proposed, it would be important to incorporate the voices of patients and health professionals to identify which recommendations are priorities for them [35]. Those preferences might be studied from a qualitative or quantitative approach, and in both cases, would be an essential research contribution.

## Data Availability

The datasets generated and/or analysed during the current study are available at https://datastudio.google.com/s/lvHGAU5AILA [27].

## Abbreviations

CMoH: Colombian Ministry of Health
CPG: Clinical practice guideline
EPOC: Effective Practice and Organisation of Care
HSG: Health systems guideline
ICT: Information and communication technology
WHO: World Health Organization
